# A human body physiological feature selection algorithm based on filtering and improved clustering

**DOI:** 10.1371/journal.pone.0204816

**Published:** 2018-10-31

**Authors:** Bo Chen, Jie Yu, Xiu-e Gao, Qing-Guo Zheng

**Affiliations:** 1 College of Mechanical and Electronical Engineering, Lingnan Normal University, Zhanjiang, China; 2 College of Information Engineering, Dalian University, Dalian, China; 3 College of Information Engineering, Lingnan Normal University, Zhanjiang, China; Liverpool John Moores University, UNITED KINGDOM

## Abstract

**Research:**

The body composition model is closely related to the physiological characteristics of the human body. At the same time there can be a large number of physiological characteristics, many of which may be redundant or irrelevant. In existing human physiological feature selection algorithms, it is difficult to overcome the impact that redundancy and irrelevancy may have on human body composition modeling. This suggests a role for selection algorithms, where human physiological characteristics are identified using a combination of filtering and improved clustering. To do this, a feature filtering method based on Hilbert-Schmidt dependency criteria is first of all used to eliminate irrelevant features. After this, it is possible to use improved Chameleon clustering to increase the combination of sub-clusters amongst the characteristics, thereby removing any redundant features to obtain a candidate feature set for human body composition modeling. Method

We report here on the use of an algorithm to filter the characteristic parameters in INBODY770 (this paper used INBODY 770 as body composition analyzer.) measurement data, which has three commonly-used impedance bands (1 kHZ, 250 kHZ, 500 kHZ). This algorithm is able to filter out parameters that have a low correlation with body composition BFM. The algorithm is also able to draw upon improved clustering techniques to reduce the initial feature set from 29 parameters to 10 parameters for any parameters of the 250 kHZ band that remain after filtering. In addition, we also examined the impact of different sample sizes on feature selection.

**Result:**

The proposed algorithm is able to remove irrelevant and redundant features and the resulting correlation between the model and the body composition (BFM which is a whole body fat evaluation can better assess the body's overall fat and muscle composition.) is 0.978, thereby providing an improved model for prediction with a relative error of less than 0.12.

## Introduction

To a certain extent, changes in the body composition reflect the change of the physical health status. In view of that the current human body composition model is constructed in the experience prediction model of body composition based on the various bioelectrical impedance measurement method (BIA), and the INBODY770 will be used as as body composition analyzer and a source of data in this paper.[1]

The equilibrium state of a human body’s composition plays an important role in maintaining the stability of the body as an environment, which is an important factor affecting human health [2]. When disease occurs, changes in human body composition tend to appear earlier than the clinical symptoms of the disease [3]. This being the case, changes in human body composition are open to being used to make correlation predictions about Hypertension, Dyslipidemia, metabolic syndrome and other diseases [4][5][6]. However, there are many relevant parameters that might influence human body composition. In addition, there can be significant nonlinearity, redundancy and irrelevance amongst the parameters [7][8][9]. There is therefore a need to reduce the amount of high-dimensional data present in human body composition parameters. Clustering methods can divide body composition parameter data into several groups or clusters so that intra-cluster objects have high degrees of similarity [10], thereby effectively sifting out redundant features according to the distance between each cluster and a central point. At the same time, it is also important to reduce the number of features and eliminate any properties that are not relevant before high-dimensional data analysis of a body’s composition can take place.

There are two ways to reduce the number of features: one is feature selection, and the other is feature extraction. Feature extraction involves some kind of combination of primitive whole features. Feature selection, however, involves choosing highly correlative features that can lead to the separation of categories from amongst the primitive whole features [11]. During measurement of human body composition it is important to reduce the impact of any irrelevant parameters that may be present amongst the large number of characteristic parameters. Feature selection algorithms are able to select a set of subsets from existing feature sets, thereby reducing the number of possible dimensions. They have attracted considerable attention in academic circles because they offer the twin advantages of simple structure and good algorithmic performance. Within feature selection itself there are two main approaches to selection: filter feature selection; and wrapper feature selection [12][13]. Wrapper selection makes direct use of characteristic classification performance as a means of evaluating the relative importance of different characteristics. This helps with the identification of key features, but the evaluation strategy involves iterative calculations by the classifier, making it slow. As a result the wrapper approach cannot generally be used for high-dimensional data sets. Filter feature selection algorithms calculate the implicit information in a feature by means of a distance measurement, an information measurement, and a dependency measurement. Depending on the calculated results of the criteria, each characteristic parameter is given a weight value and important characteristics are then selected according to their weight. Although the efficiency of filter algorithms is high [12], the method does not take full account of any possible redundancy between characteristic parameters. As a result selected parameter subsets are likely to contain large amounts of redundancy. So it can be seen that both the wrapper and filter feature selection methods have their own advantages and disadvantages.

In recent years, feature selection algorithms that combine both approaches been proposed. In [13] a ReliefF algorithm is first of all used to filter out irrelevant features. Correlation analysis and a sequential backward search wrapper algorithm is then used to remove redundant features. This method offers better overall performance, but it is not suitable for large-scale datasets because of its high algorithmic complexity. In [14] the authors propose a maximum correlation and minimum redundancy filter-wrapper hybrid feature selection method that is based on Particle Swarm Optimization (PSO). By fusing the filter into the wrapper and making use of both the high efficiency of the filter and the high accuracy of the wrapper, this method is able to improve the speed and performance of the search. Thus, this hybrid model can improve the convergence speed of a PSO algorithm whist optimizing the character subset. In [15] a two-step strategy is proposed that uses both the filter and wrapper methods to achieve feature selection by combining information gain and harmony search algorithms to select the emotional characteristics of speech. A Support Vector Machine (SVM) is used here as the classifier for speech feature selection. The advantage of this approach is that it reduces the scale of the optimized subset and thus the running time. The method suggested in [16] first of all uses filter criteria to rank features. The classifier's performance is then used as the basis of the fitness function. In this case a genetic algorithm (GA) is used to search for character subsets. When compared to single filter or wrapper methods, this algorithm is able to improve both the performance and the efficiency of feature selection. In [17] four different filter methods are used to initialize GA populations. Having done this, the classification accuracy of the neural networks is used to evaluate the feature subset. This hybrid character selection model effectively reduces the size of the feature subset and improves the classification recognition accuracy. However, the computational complexity of the wrapper model increases exponentially as the number of dimensions increases. As a result, this combination algorithm cannot be used for high-dimensional datasets.

In the domain of data mining, clustering theory has recently started to be used for feature selection algorithms [18]. For instance, in [19] a Clearness-Based Feature Selection (CBFS) method, based on clustering, is used to obtain a candidate character set by iterating clustering results. In [20] the authors use a Fuzzy Self-Constructing Feature Clustering (FSFC) approach that is based upon feature average correlation. Here, features with a strong correlation are assembled in the same cluster by using a clustering algorithm. After this representative characteristics from each cluster are selected to form an optimal character set, thereby eliminating redundant and irrelevant features. In [21] a weighted feature selection algorithm facing to clustering (ENFSA) is proposed still shows good performance in datasets with a larger number of feature dimensions when compared to clustering. Overall, clustering algorithms have the disadvantage of not being very effective at removing irrelevant features. In this paper we therefore make use of a feature selection algorithm that combines filtering criteria and clustering, with a Filter Criterion method being used to remove irrelevant physiological characteristics, and a clustering algorithm being used to remove redundant features. In our view this best meets the requirements of human body composition modeling.

The main contributions in this paper are conclude as follows:

- A feature filtering method based on Hilbert-Schmidt dependency criteria is first of all used to eliminate irrelevant features.
- In this paper, we using improved Chameleon clustering to increase the combination of sub-clusters amongst the characteristics, thereby removing any redundant features to obtain a candidate feature set for human body composition modeling.
- We report here on the use of an algorithm to filter the characteristic parameters in INBODY770 measurement data, which has three commonly-used impedance bands (1 kHZ, 250 kHZ, 500 kHZ). This algorithm is able to filter out parameters that have a low correlation with body composition BFM.
- The overall goal of this paper is to develop a method for overcoming the impact that redundancy and irrelevancy may have on human body composition modeling. SectionⅡ introduces filtering using Hilbert-Schmidt Dependency Criterion which has been shown to remove features that are not related to the class. Section Ⅲ presents improved Chameleon clustering was used for the purposes of feature selection and optimization. This is highly effective for the elimination of redundant features. Section Ⅳ presents experimental results and use an algorithm to filter the characteristic parameters in INBODY770 measurement data. Finally Section Ⅴ concludes the paper.

## Using the Hilbert-Schmidt Dependency Criterion (HSIC) feature filtering method

Feature filtering selection uses distance, information, dependency and consistency measures as evaluation criteria to evaluate each feature in a feature set. According to the results of this evaluation the features are then sorted. Finally, those with preferred performance characteristics are selected, thereby eliminating any irrelevant features. The evaluation criteria play an important role in feature filtering selection algorithms, and their choice directly affects the final performance of the algorithm. Hilbert-Schmidt Dependency Criterion (HSIC) is a kernel-based independent measure, where the cross covariance operator is defined in the reproduction of a Hilbert space. The criterion of independence is obtained using empirical estimation of the operator norm, which can be used to measure the similarity between the two data distributions. It is therefore widely used in feature selection to reduce dimensions [22][23] As human body characteristics and body composition indices have some dependence it is possible to use HSIC to quantify the correlation between the body characteristic parameters and body composition categories.

Given the human body characteristic parameter set space *F*, we can define a non-linear feature map ϕ:F→F. The feature points *f*_1_,*f*_2_,⋯,*f*_*m*_ can then be mapped into a reproducing kernel Hilbert space F, so the kernel function can be written as:

$$k(x,x^{\prime })={\langle \phi (x),\phi (x^{\prime })\rangle }_{F},x,x^{\prime }\in F$$ (1)

The equation: ${\langle •,•\rangle }_{F}$ is an inner product in the space F. Similarly, an individual component class mapping ψ:C→C can be defined, The body composition index *C* space can then be mapped into the reproducing Hilbert space, denoted as C, with the corresponding kernel function being as follows:

$$l(y,y^{\prime })={\langle \psi (y),\psi (y^{\prime })\rangle }_{C},y,y^{\prime }\in C$$ (2)

The kernel function can calculate the inner product between two feature points in a feature space projection without explicitly calculating the specific mapping φ and incurring any computational cost related to the number of dimensions. Thus, the cross covariance operator of a feature and the body class can be defined as:

$$C_{fc}=E[(\phi (f)-\mu_{f})⊗(\psi (c)-\mu_{c})]$$ (3)

In the above formula, ⊗ represents the tensor product, and $\mu_{f}=E_{f}[\phi (f)]$ and $\mu_{c}=E_{c}[\phi (c)]$ indicate expectations [24]. The norm ${\left\|C_{fc}\right\|}_{HS}^{2}$ of the square of this covariance is called HSIC. The expression of this is [25]:

$$HSIC(F,C,{\mathbb{P}}_{FC})={\left\|C_{fc}\right\|}_{HS}^{2}=E_{ff^{\prime }cc^{\prime }}[k(f,f^{\prime })l(c,c^{\prime })]+E_{ff^{\prime }}[k(f,f^{\prime })]E_{cc^{\prime }}[l(c,c^{\prime })]-2E_{fc}[E_{f^{\prime }}[k(f,f^{\prime })]E_{c^{\prime }}[l(c,c^{\prime })]]$$ (4)

In the collected data set *T* = (*O*,*F*,*C*), the data sample set *O* = {*o*_1_,*o*_2_,⋯,*o*_*n*_}, and the selected feature set *F* = {*f*_1_,*f*_2_,⋯,*f*_*m*_}, somatic classification is based on C = {*c*_1_,*c*_2_,⋯,*c*_*n*_}. For a feature f and a body composition category c, the greater the value of HSIC, the greater the dependency of c on f, when the value of HSIC is equal to 0, this indicates that c and f have no relevance at all.

## A human body feature clustering method based on improved Chameleon clustering

**Fig 1:**
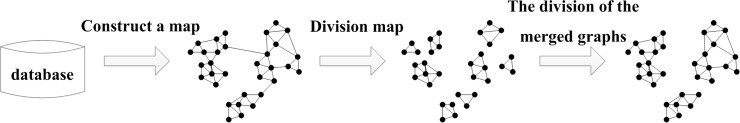
Outline view of the Chameleon clustering algorithm.

Clustering can divide a data object into multiple subgroups or sub-clusters, so that the objects within the sub-cluster have a high similarity 26. If a feature is taken as the object for clustering, sub-clusters can be divided as needed and the distance between each feature and the center point calculated. In the same sub-cluster, in order to reduce redundancy, features that are far from the center can be filtered. In this paper a Chameleon hierarchical clustering method is used for the initial selection of feature parameters. Chameleon uses a cohesive hierarchical clustering method to construct a feature sparse graph according to the K-Nearest Neighbor. Each vertex in the graph represents a data object, with an edge between the two vertices, and where the weight of the edge can reflect the similarity between objects [26][27]. The principle of this algorithm is shown in Fig 1:

Similarity amongst the characteristics of sub-clusters is based on two points: 1) the interconnection of the objects in the sub-cluster; 2) the proximity of the sub-cluster. If two feature sub-clusters have a high degree of interconnection and are close to each other, widely separated feature sub-clusters will be replaced by mergers. The similarity between two features is determined according to the relative connectivity degree RI and the relative proximity degree RC of the two feature clusters [28]. The calculation method can be summarized as follows: There is a characteristic data set *F* = {*f*_1_,*f*_2_,⋯,*f*_*m*_}, which is normalized and filtered by a filter. The data set cluster *F* is divided into sub-clusters *f*_1_ and *f*_2_, which divide *F* into *f*_1_ and *f*_2_ The edge of the cut then has the least weight and the relative interconnection between the characteristic sub-clusters *f*_1_ and *f*_2_ is greater. The relative interconnection degree *RI*(*f*_1_,*f*_2_) of the two feature clusters *f*_1_ and *f*_2_ is defined as the degree of relative interconnection between the feature clusters *f*_1_ and *f*_2_. The internal interconnection of the two clusters *f*_1_ and *f*_2_ is:

$$RI(f_{1},f_{2})=\frac{\left|EC_{\left\{f_{1},f_{2}\right\}}\right|}{\frac{1}{2}(|EC{|}_{f_{1}}+|EC{|}_{f_{2}})}$$ (5)

Here, $EC_{\left\{f_{1},f_{2}\right\}}$ is the side cutting that contains the clusters of *f*_1_ and *f*_2_. Similarly, $|EC{|}_{f_{1}}\mathrm{or}|EC{|}_{f_{2}}$ is the minimum sum of the side cutting that divides *f*_1_ (or *f*_2_) into roughly equal parts.

The relative proximity *RC*(*f*_1_,*f*_2_) of the two feature clusters *f*_1_ and *f*_2_ is defined as the absolute approximation between *f*_1_ and *f*_2_. The normalization of the internal proximity of the two feature clusters *f*_1_ and *f*_2_ is:

$$RC(f_{1},f_{2})=\frac{{\bar{S}}_{EC_{\left\{f_{1},f_{2}\right\}}}}{\frac{\left|f_{1}\right|}{\left|f_{1}|+|f_{2}\right|}{\bar{S}}_{EC_{f_{1}}}+\frac{\left|f_{1}\right|}{\left|f_{1}|+|f_{2}\right|}{\bar{S}}_{EC_{f_{2}}}}$$ (6)

**Fig 2:**
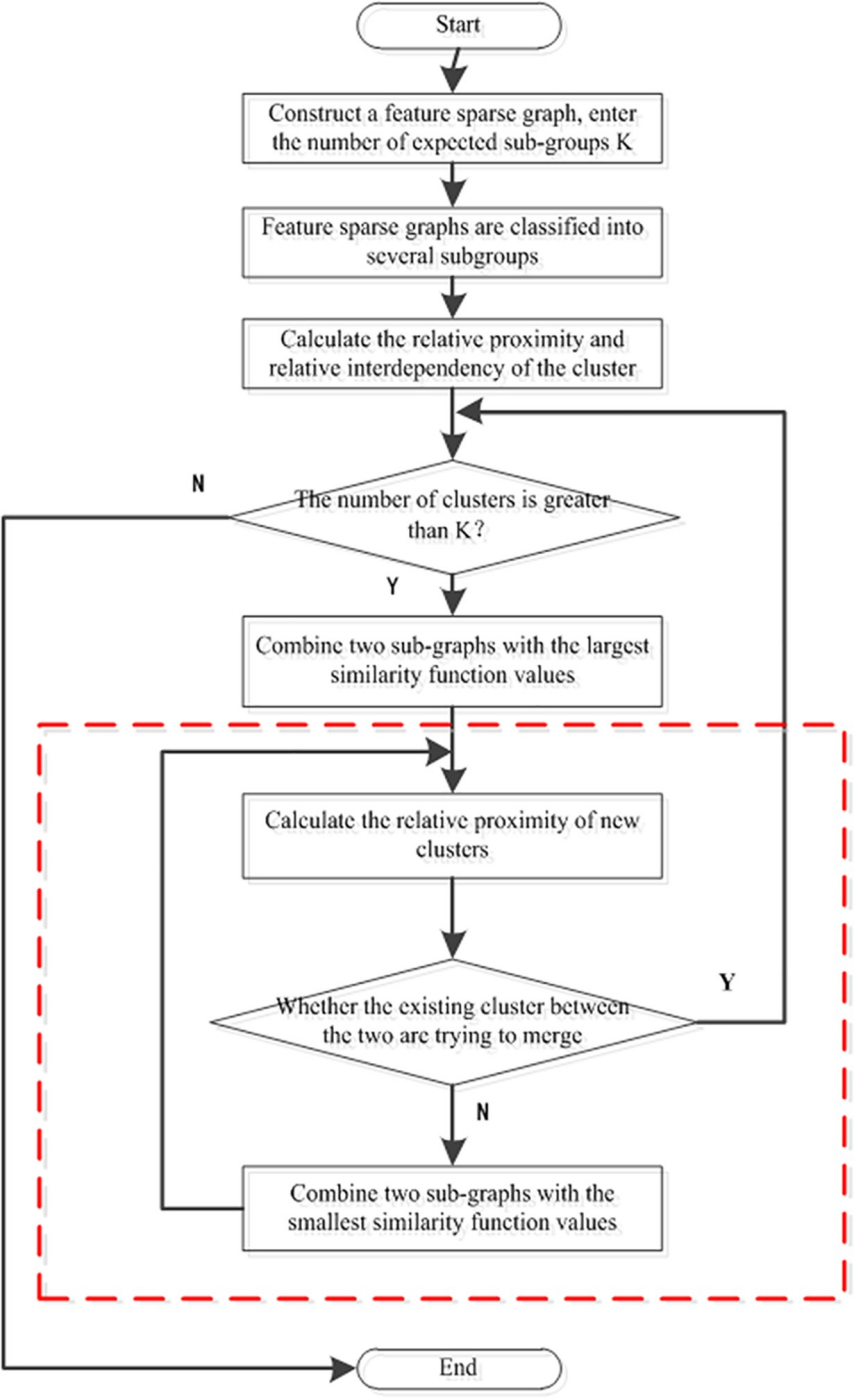
Schematic diagram of the improved Chameleon algorithm.

Here, ${\bar{S}}_{EC_{\left\{f_{1},f_{2}\right\}}}$ is the average weight of the edge connecting *f*_1_’s vertex and *f*_2_’s vertex. ${\bar{S}}_{EC_{f_{1}}}(\mathrm{or}\;{\bar{S}}_{EC_{f_{2}}})$ is the average weight of the edge of the smallest dichotomy *f*_1_ (or *f*_2_). The similarity between two sub-clusters is determined by the relative interconnection and the relative approximation of the characteristic sub-clusters *f*_1_ and *f*_2_, and the two sub-clusters with the largest similarity are selected to be combined [29][30]. However, because the division of the clustering in a Chameleon algorithm is only based on the number of sub-graphs, it is impossible to combine the traversal attempts of all the feature sub-clusters. Therefore, we offer here an improved algorithm, as shown in Fig 2 (with the improvement in the dotted box):

As can be seen, by evaluating the quality of feature selection after the consolidation of sub-clusters and attempting to merge between two existing clusters, it is possible to effectively remove redundant features, which guarantees the clustering effect of the algorithm overall.

## Combing improved clustering and filter criteria for human feature selection algorithms

**Fig 3:**
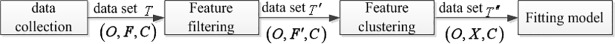
Body feature parameter selection process.

In this paper, a feature filtering selection algorithm is first of all used to remove features that are not related to a body composition category. After this, the M-Chameleon feature clustering method is used to remove redundant features, As a result, the advantages of both feature filtering selection algorithms and feature clustering algorithms are maximized. The process for feature filtering selection is shown in the Fig 3:

Let us take a collected data set *T* = (*O*,*F*,*C*), a data sample set *O* = {*o*_1_,*o*_2_,⋯,*o*_*n*_}, a feature selection set *F* = {*f*_1_,*f*_2_,⋯,*f*_*m*_}, and a body composition category C = {*c*_1_,*c*_2_,⋯,*c*_*n*_}. The C of some specific body composition category as taken as input, the features that are not related to the C of the body composition category are removed by feature filtering, resulting in an initial feature set *F*′ = {*f*_1_,*f*_2_,⋯,*f*_*h*_}. This forms the initial data set *T*′ = (*O*,*F*′,*C*). To cluster the features of the initial data set, features where the distance between similar feature clusters is at its greatest are merged and replaced. In this way different feature clusters are eventually formed, with the number l. A maximum HSIC value is then selected from each cluster as a representative feature to make up a feature set *X* = {*f*_1_,*f*_2_,⋯,*f*_*l*_}, thereby arriving at the selected physiological characteristics of the human body. The specific algorithmic process is as follows:

Input: the feature set *F* = {*f*_1_,*f*_2_,⋯,*f*_*m*_}, the data sample set *O* = {*o*_1_,*o*_2_,⋯,*o*_*n*_}, the body composition category set *C*.

Output: *X* is used to predict the optimal feature set of the body composition class *C*.

Step 1: The initial feature set *F*′ and the final optimal subset *X* are initialized to an empty set, denoted as *F*′ = ∅, *X* = ∅.

Step 2: For each feature {*f*_1_,*f*_2_,⋯,*f*_*m*_} ∈ *F*, the HSIC value of the body composition category *C* is computed, which represents the correlation between the physical characteristics and the body composition category.

Step 3: Rank the feature *F* = {*f*_1_,*f*_2_,⋯,*f*_*m*_} from big to small according to the HSIC value.

Step 4: Add the features at the top of K to the feature set *F*′, then form the initial feature set *F*′ = {*f*_1_,*f*_2_,⋯,*f*_*h*_} and the initial data set *T*′ = (*O*,*F*′,*C*).

Step 5: The features sparse graph *G* = (*F*′,RI,RC) is constructed according to the data *T*′. *F*′ is the initial feature set, RI is an edge set connecting the features to one another, RC is the similarity between features. The initially expected number of clusters is k, which is *G* = {*G*_1_,*G*_2_,⋯,*G*_*k*_}.

Step 6: Calculate the distance between the clusters and rank them, then judge whether the number of sample clusters h is equal to the number of initially expected clusters k.

Step 7: If the value is not equal, select only the two largest clusters subjected to the similarity function to merge. Set h = h-1. If the value is equal, end the process.

Step 8: Recalculate the relative proximity RC of the new cluster, traverse all sub-clusters, and within all of the sub-clusters try to merge between the two established clusters.

Step 9: Assess whether all of the sub-clusters can be merged. If this is true, return to step 6; otherwise, merge the two sub-clusters where the similarity function is the smallest, then return to step 8.

Step 10: Choose the feature with the maximum HSIC value from each feature cluster to form the optimal feature set X.

## Experimental analysis

### The experimental data

For our experimental analysis, the body composition data measured by INBODY770 was used as the data set (the data are from 18–65 years old adults measured by INBODY770 in a hospital in Beijing. 100 samples were randomly selected for data training), denoted here as *T* = (*O*,*F*,*C*). This includes the set of parameters that have the most important influence on body composition, such as weight, height, age, and sex. Impedance values for each segment of the human body were used as the first characteristic parameters. The reciprocal of each segment of impedance 1/*R*_*i*_, the square of each segment of impedance *R*_*i*_^2^, and the product of the two numbers *R*_*i*_*R*_*j*_ were used as additional characteristic parameters. INBODY770 has three measurement bands, 1 kHZ, 250 kHZ and 500 kHZ (For our experimental analysis, the body composition data measured by INBODY was used as the data set. the human body composition analyzer (Inbody770) uses multi-frequency multi-segment measurement methods for research. This manuscript selects the upper and lower limits of the frequency band and the median, more representative frequency bands for research and analysis.). In this paper we will discuss the relationship between body composition and the characteristic parameters across all three bands using different sample rates. For analysis purposes our first characteristic parameter was *R*_1_,*R*_2_,*R*_3_,*R*_4_,*R*_5_,*A*,*H*,*W* (*R*_1_ is left upper limb impedance, *R*_2_ is right upper limb impedance, *R*_3_ is the trunk impedance, *R*_4_ is left lower limb impedance, *R*_5_ is right lower limb impedance, *A* is age, *H* is height, *W* is weight) and the second characteristic parameter we selected was 1/*R*_1_, 1/*R*_2_, 1/*R*_3_, 1/*R*_4_, 1/*R*_5_, *R*_1_*R*_2_, *R*_1_*R*_3_, *R*_1_*R*_4_, *R*_1_*R*_5_, *R*_2_*R*_3_, *R*_2_*R*_4_, *R*_2_*R*_5_, *R*_3_*R*_4_, *R*_3_*R*_5_, *R*_4_*R*_5_, *R*_1_^2^,*R*_2_^2^,*R*_3_^2^,*R*_4_^2^,*R*_5_^2^. The first characteristic parameter and the second characteristic parameter of the initial feature set were denoted as *F* = {*f*_1_,*f*_2_,⋯,*f*_*m*_}. The body composition category set *C* included Body Fat Content (BFM) and Total Body Water (TBW). For more details about the sample data see the attachment file named Inbody Excel Data.

### Experimental results and discussion

**Fig 4:**
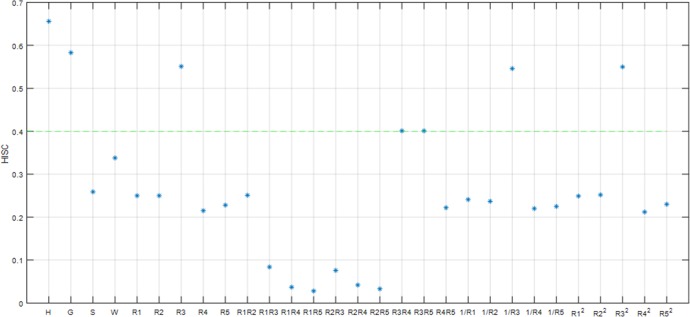
The correlation between the characteristic parameters calculated by the filtering algorithm within the 1 kHZ band.

**Fig 5:**
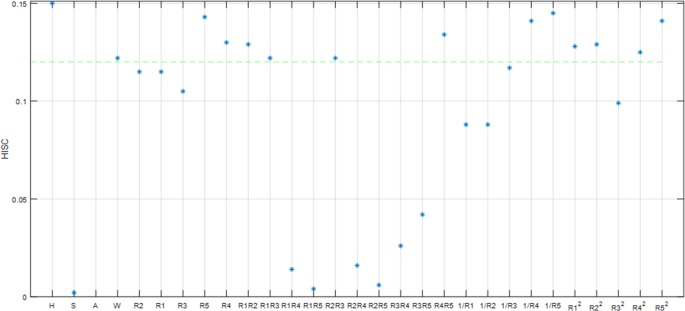
The correlation between the characteristic parameters calculated by the filtering algorithm within the 250 kHZ band.

**Fig 6:**
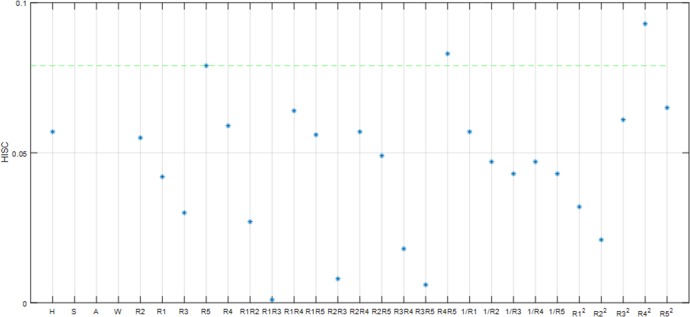
The correlation between the characteristic parameters calculated by the filtering algorithm within the 500 kHZ band.

Using the original feature set *F* = {*f*_1_,*f*_2_,⋯,*f*_*m*_}, the data sample set *O* = {*o*_1_,*o*_2_,⋯,*o*_*n*_}, and human body composition BFM and TBW, we ran a filtering algorithm using SPSS for a 100 person sample. The following Figs 4 and 5 and 6 show characteristic parameter correlations after filtering the body composition BFM and TBW.

It can be seen from the Fig 4, Fig 5, Fig 6 that as the impedance band is gradually increased, the value of the impedance decreases, and the BFM information contained in each characteristic parameter also reduces gradually. Using a confidence interval of 80% as the filter, we selected the characteristic parameters. The results of this are shown in Table 1 below:

**Table 1:** Features after running the filter algorithm in different frequency bands.

Frequency Bands	Category	Primitive Feature Set *F F*	Filtered Characteristics *F*'	Feature Set
1 kHZ	Body Fat (BFM)	29	7	*G*,*H*,1/*R*_3_,*R*_3_^2^,*R*_3_,*R*_3_*R*_4_,*R*_3_*R*_5_
250 kHZ	Body Fat (BFM)	29	14	*R*_4_^2^,*R*_5_^2^,*R*_2_^2^,*R*_1_^2^,*R*_4_*R*_5_,*R*_1_*R*_2_,*R*_4_,*R*_5_,*A*,*H*
500 kHZ	Body Fat (BFM)	29	6	*G*,*A*,*W*,*R*_4_^2^,*R*_5_,*R*_4_*R*_5_

As Table 1 demonstrates, the filtering algorithm greatly reduces the number of original feature sets, but the 250 kHZ frequency band produces more clustering. Therefore, the characteristics within the intermediateimpedance band 250 kHZ were selected for clustering analysis after they had been filtered and the redundant information removed.

**Fig 7:**
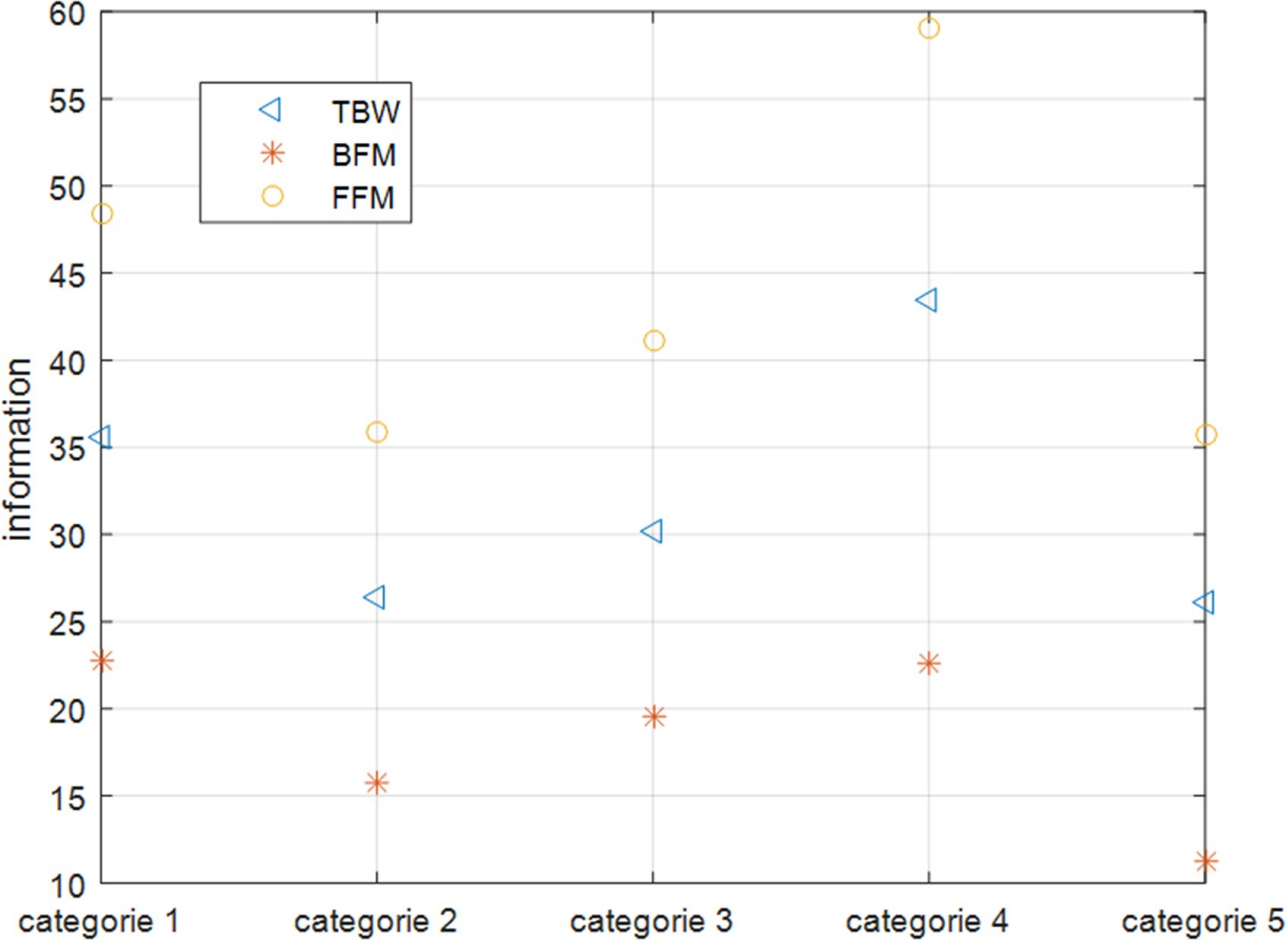
Analysis of the number of clustered parameters after using the filter algorithm. The legend for Fig 7 is” TBW, BFM, FFM”.

According to the Fig 7, we can see that the body composition parameters TBW, BFM and FFM (Fat Free Mass) contain more information when they are clustered into four categories. Therefore, clustering analysis was carried out using four types of characteristic parameters after the filtering results had been screened. In order to study the impact of different sample sizes on the screening of feature parameters, we used different experimental samples containing 20, 40, 60, 80 and 100 people. The samples were then divided into 4 categories to conduct the clustering analysis, with BFM as the clustering center.

**Fig 8:**
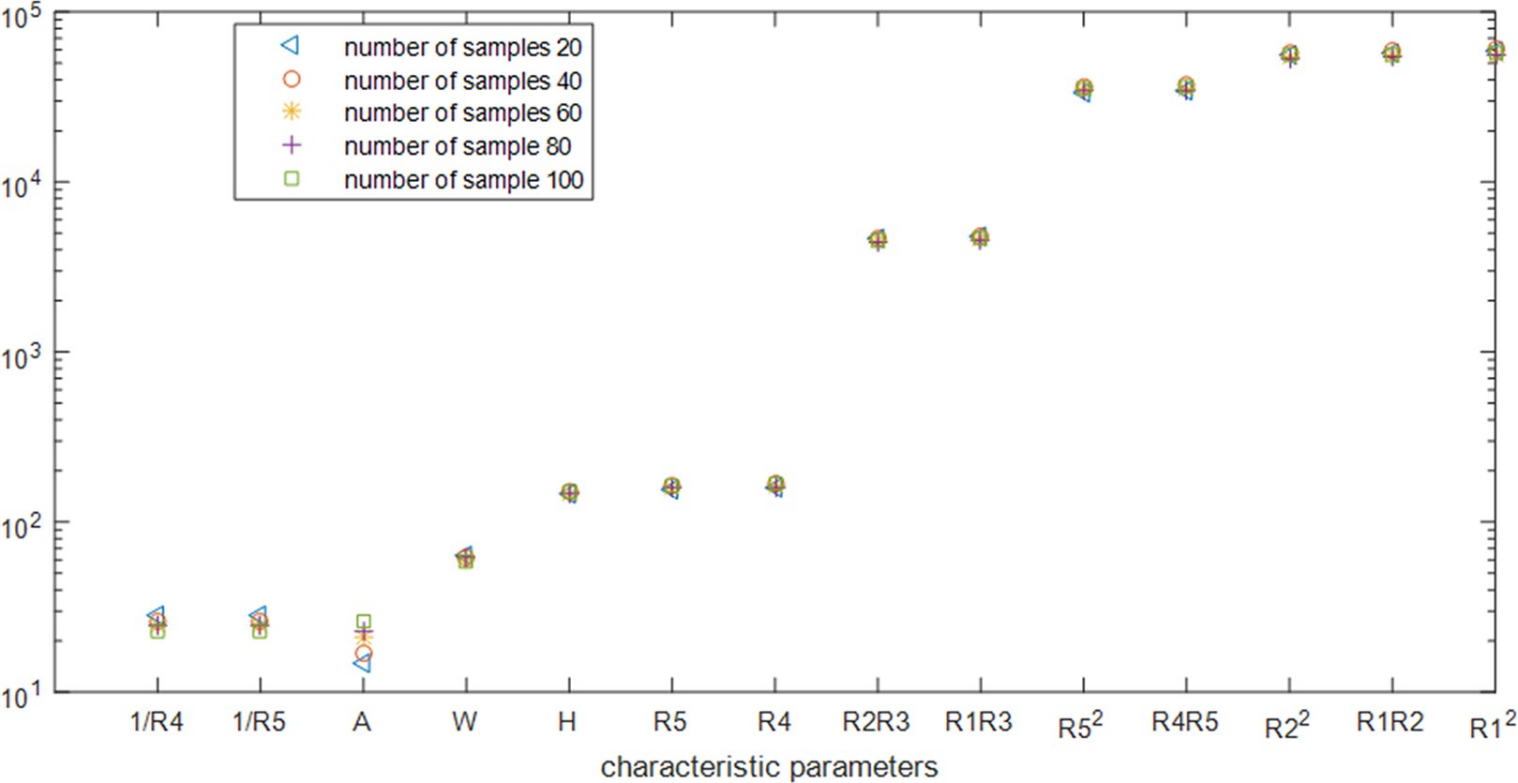
Distance between the characteristic parameters and the BFM index when the number of samples is split into four categories.

As can be seen from Fig 8, across all sample sizes, only the clustering for age shows any significant change. 1/*R*_4_, 1/*R*_5_ assemble into one class, *R*_4_, *R*_5_, A, H, W assemble into one class, *R*_4_*R*_5_, *R*_1_*R*_2_, *R*_2_^2^, *R*_*1*_^*2*^, *R*_5_^2^ assemble into one class and *R*_2_*R*_3_, *R*_1_*R*_3_ assemble into one class. After the characteristic parameters obtained by the filter algorithm are clustered into 4 categories, 1/*R*_4_, *R*_4_, *R*_*1*_^*2*^, *R*_1_*R*_3_ those that are far from the cluster center BFM can be removed. Table 2 shows the selection of feature parameters after running the filtering and clustering algorithms.

The legend for Fig 8 is “number of samples 20, number of samples 40, number of samples 60, number of samples 80, number of samples 100”

**Table 2:** The characteristics of the parameters after using the filtering and improved clustering algorithms.

Frequency Band	Category	The Characteristics of the Algorithm X	Feature Set
250 KHZ	Body Fat (BFM)	10	A,H,W,R5,R1R2,R2R3,R4R5,1/R5, R22, R52

Table 3 lists the candidate feature sets and time complexity for the prediction of body composition BFM using the three feature selection methods:

**Table 3:** Comparison of optimal feature sets and complexity.

Number	Algorithm Used	Characteristic Clustering X	Time Consumption (s)	Feature Set
1	mRMR	10	3.2	W,S, A, R3, 1/R2, 1/R1, 1/R3, R42, R4R5, R52
2	Filter & Wrapper	9	2.9	1/R3,W,S, R22, R42, R4R5, R52, 1/R1, R5
3	This Paper	10	2.8	A,H,W,R5,R1R2,R2R3,R4R5,1/R5, R22, R52

It can be seen from Table 3 that the size and time complexity of the candidate feature set obtained by using our own algorithm are smaller than they are for the Filter and Wrapper, and MRMR feature selection algorithms when the data set has the same overall size.

In order to verify the performance of the feature selection algorithm. For body composition (BFM), Using the MRMR, Filter and Wrapper combined feature selection algorithms, the paper’s algorithm to select the candidate parameters. What's more, in order to accurately measure the advantages and disadvantages of candidate feature set (in the case of a given body composition BFM), we will sample the first 80 as a training sample set, recorded as *T*_2_ = {(*x*_81_,*y*_81_),(*x*_82_,*y*_82_),⋯,(*x*_100_,*y*_100_)}, the last 20 as a test sample, recorded as *T*_2_ = {(*x*_81_,*y*_81_),(*x*_82_,*y*_82_),⋯,(*x*_100_,*y*_100_)}, and *x*_*i*_ ∈ *R*^*l*^ is setting as the input characteristic parameter value, as an independent variable, *y*_*i*_ ∈ *R* is the actual body composition value, as the dependent variable; Use multiple linear regression in SPSS software to train *T*_1_. Table 4 shows the model summary for regression modeling of BFM using the above feature set:

**Table 4:** Model summary.

Model	R	R Side	Adjusted R Side	Standard Estimation Error
W,S, A, R3, 1/R2, 1/R1, 1/R3, R4^2, R4R5, R5^2	0.927^a^	0.859	0.843	2.6173
1/R3,W,S, R22, R4^2, R4R5, R5^2, 1/R1, R5	0.906^b^	0.821	0.803	2.9340
A,H,W,R5,R1R2,R2R3,R4R5,1/R5, R2^2, R5^2	0.978^c^	0.957	0.953	1.4399

a. Predictor: (constant), W,S, A, R3, 1/R2, 1/R1, 1/R3, R4^2, R4R5, R5^2

b. Predictor: (constant), 1/R3,W,S, R2^2, R4^2, R4R5, R5^2, 1/R1, R5

c. Predictor: (constant), A,H,W,R5,R1R2,R2R3,R4R5,1/R5, R2^2, R5^2

According to Table 4, we can see that the correlation between the physiological feature set and BFM in models 1, 2 and 3, the correlation is 0.927, 0.906, 0.978, therefore the feature set obtained by using this paper algorithm has the strongest correlation with the body composition.

A predictive model was used to predict a test set *T*_2_ that could be compared with the actual value. A comparison map between the predicted value and the actual value for the BFM model and the error analysis was thus obtained.

According to the obtained regression coefficients of each model, the prediction equations are as listed below:

$$\begin{array}{l} BFM_{1}=0.041*W+0.126*S+0.523*A-0.212*R_{3}+0.171*1/R_{1}+0.126*1/R_{2}+0.179*1/R_{3}+1.132*R_{4}{}^{2}+0.13R_{4}R_{5}+0.127*R_{5}{}^{2}-8.56\\ BFM_{2}=0.313*W-0.044*S-0.125*1/R_{3}+0.108*1/R_{1}+0.016*R_{4}{}^{2}-0.01R_{2}{}^{2}+0.071R_{5}{}^{2}+0.072*R_{4}R_{5}-0.526R_{5}+5.674\\ BFM_{3}=0.464*A-0.15*H+0.122*W-0.143*R_{5}+0.129*R_{1}R_{2}+0.122*R_{3}R_{2}-0.134*R_{4}R_{5}+0.145*1/*R_{5}+0.129*R_{2}{}^{2}-0.141R_{5}{}^{2} \end{array}$$

**Fig 9:**
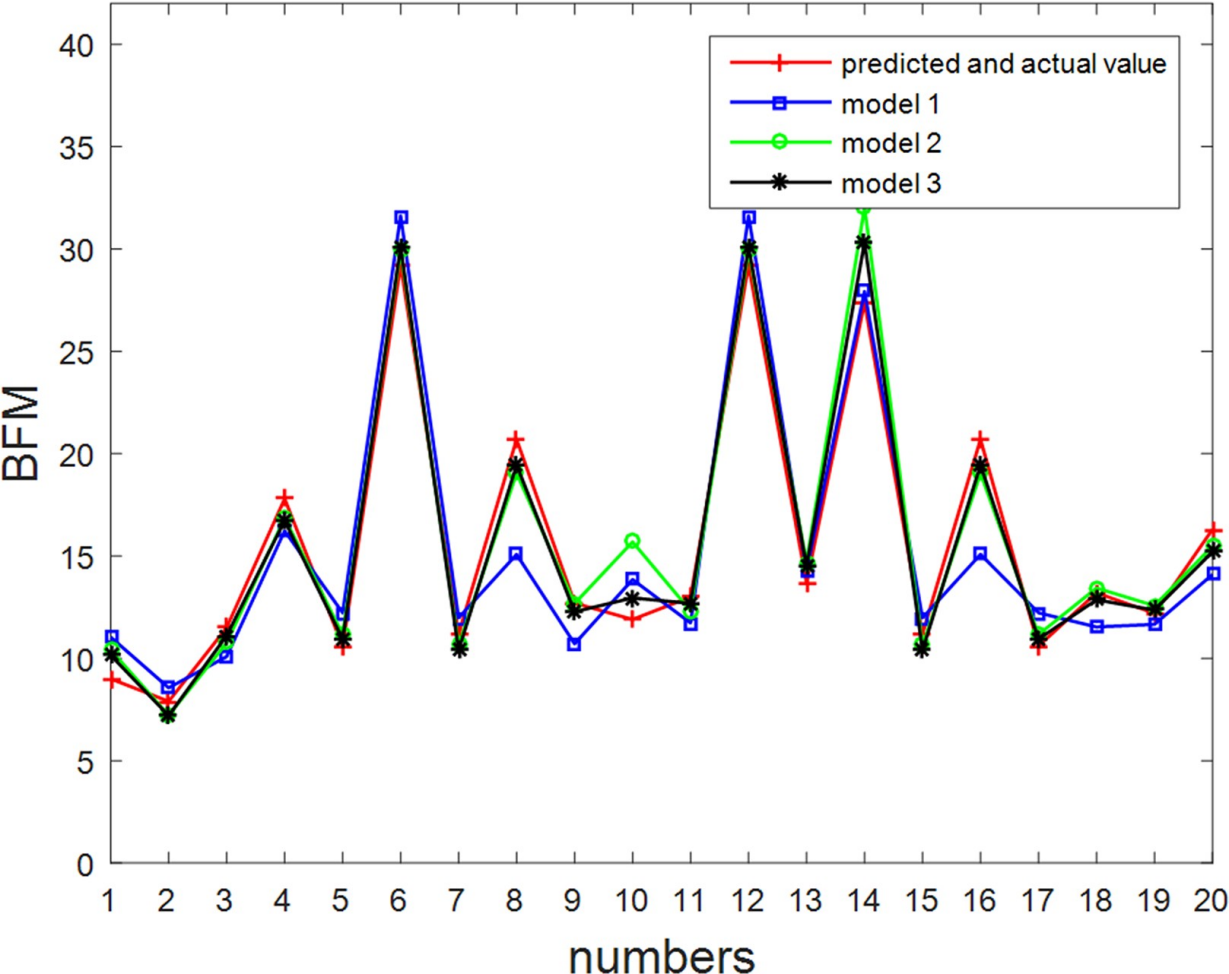
Comparison of predicted and actual values for the BFM model. The legend for Fig 9 is “predicted and actual values, model 1, model 2, model 3”.

**Fig 10:**
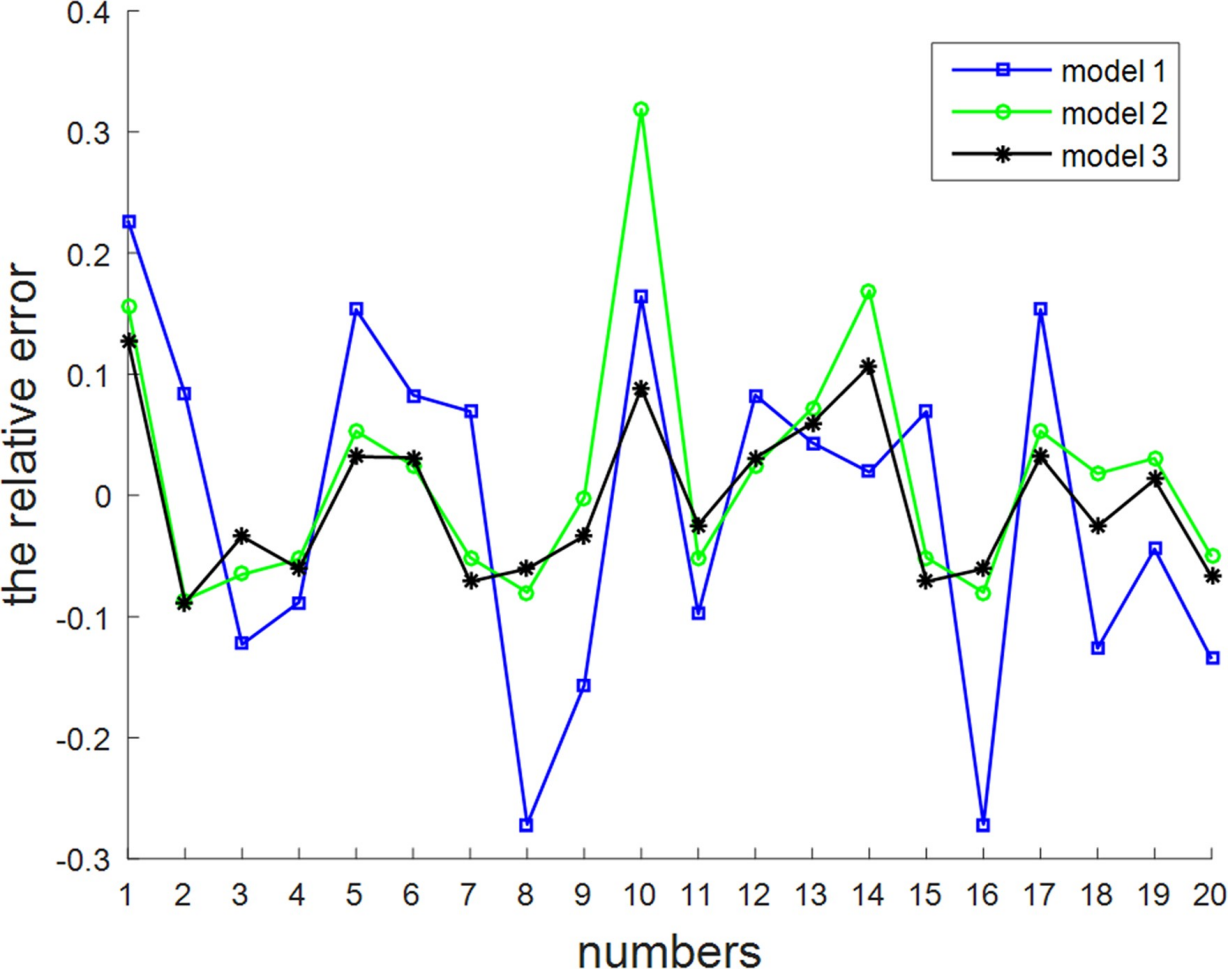
Comparison of the relative error for the predicted values of the BFM model. The legend for Fig 10 is “model 1, model 2, model 3”.

It can be seen from Fig 9 and Fig 10 that the prediction model for feature construction using this paper’s algorithm is more effective, whilst the relative error is less than 0.12. The results show that the feature set and body composition model obtained according to a human body physiological feature selection algorithm that is based on filtering and improved clustering can provide good correlative performance. This in turn can improve the fitting precision and reduce the prediction error for body composition prediction models.

## Conclusion

The body composition model is closely related to the physiological characteristics of the human body. Filtering using Hilbert-Schmidt Dependency Criterion has been shown to remove features that are not related to the class. In existing human physiological feature selection algorithms, it is difficult to overcome the impact that redundancy and irrelevancy may have on human body composition modeling. Improved Chameleon clustering was used for the purposes of feature selection and optimization. This is highly effective for the elimination of redundant features. An optimal feature parameter set for constructing a component model was selected that solved the problem of there being both too many human physiological characteristic parameters and too much redundancy within them. BFM as a whole body fat evaluation can better assess the body's overall fat and muscle composition. BFM is measured by the method of physiological electrical impedance analysis (BIA), what’s more, the BIA measurement is safe, easy to operate, and widely used in human body composition analysis.
